# Evaluation of Functional NK Cell Responses in Vaccinated and SIV-Infected Rhesus Macaques

**DOI:** 10.3389/fimmu.2016.00340

**Published:** 2016-08-31

**Authors:** Diego A. Vargas-Inchaustegui, Olivia Ying, Thorsten Demberg, Marjorie Robert-Guroff

**Affiliations:** ^1^Vaccine Branch, Center for Cancer Research, National Cancer Institute, National Institutes of Health, Bethesda, MD, USA

**Keywords:** NK cell, SIV, rhesus macaque, cytokine, cytotoxicity

## Abstract

NK cells are crucial components of the innate immune system due to their capacity to exert rapid cytotoxic and immunomodulatory function in the absence of prior sensitization. NK cells can become activated by exposure to target cells and/or by cytokines produced by antigen-presenting cells. In this study, we examined the effects of a simian immunodeficiency virus (SIV) vaccine regimen and subsequent SIV infection on the cytotoxic and immunomodulatory functions of circulatory NK cells. While vaccination did not significantly impact the capacity of NK cells to kill MHC-devoid 721.221 target cells, SIV-infection led to a significant decrease in target cell killing. NK cells from uninfected macaques were responsive to a low dose (5 ng/ml) of IL-15 pre-activation, leading to significant increases in their cytotoxic potential, however, NK cells from SIV-infected macaques required a higher dose (50 ng/ml) of IL-15 pre-activation in order to significantly increase their cytotoxic potential. By contrast, no differences were observed in the capacity of NK cells from vaccinated and SIV-infected macaques to respond to IL-12 and IL-18. Similarly, NK cells both before and after infection exhibited equivalent responses to Fc-mediated activation. Collectively, our results show that early SIV-infection impairs the natural cytotoxic capacity of circulatory NK cells without affecting Fc-mediated or cytokine-producing function.

## Introduction

NK cells participate in early control of viral infection through their capacity to exert rapid cytolytic and immunoregulatory functions (1). Their functional activity is tightly controlled by the balance of inhibitory and activatory signals engaged via various cell surface receptors (2, 3). Differential expression of these inhibitory and activatory receptors gives rise to NK cell heterogeneity and allows NK cells to respond to various stimuli (4). During antiviral immune responses, NK cells can receive activatory signals through different types of toll-like receptors (TLR) and activatory killer-cell immunoglobulin-like receptors (KIRs) (5). Importantly, chronic viral infections have been shown to affect NK cell maturation at different stages and to promote the expansion of functionally impaired subpopulations (6). Specifically for human immunodeficiency virus (HIV) infection, viremia has been correlated with an expansion of CD16^+^ NK cells that exhibit anergy and decreased cytotoxic potential (7–9).

Rhesus macaques (*Macaca mulattta*) are the animal model of choice for evaluating novel HIV vaccine and therapeutic candidates through the use of the simian immunodeficiency virus (SIV) (10). As in humans, macaque circulatory NK cells can be divided into subsets based on their cell surface expression levels of CD16 and CD56. CD16^+^ NK cells comprise the major circulatory subset (~90%) and contain higher amounts of cytoplasmic granules rich in cytotoxic proteins. Moreover, CD16^+^ NK cells are capable of performing antibody-dependent NK cell responses. CD56^+^ NK cells are a smaller (~1–5%) immunoregulatory subset that is specialized in cytokine production. Unique to rhesus macaques, CD16^−^CD56^−^ double-negative NK cells represent 5–10% of circulatory NK cells and are capable of both cytotoxic and immunoregulatory functions (11–13). Similar to human HIV infection, SIV infection in macaques leads to an increase in the number of circulatory and LN-resident CD16^+^ NK cells (14, 15). Interestingly, LN-resident CD16^+^ NK cells from SIV-infected macaques displayed increased perforin expression and cytotoxicity (15). Although several studies in human HIV infection have evaluated the impact of vaccination and virus replication on the distribution and functional status of human NK cells, only a few studies have focused on vaccinated and SIV-infected rhesus macaques (16). For these reasons, in the present study, we examined the NK cell cytotoxic potential and functionality of circulatory NK cells in rhesus macaques vaccinated sequentially with SIV-specific DNA, replication-competent adenoviral recombinants, and SIV proteins, prior to and after SIV infection. Our results show that while vaccination did not significantly impact the cytotoxic potential of circulatory NK cells, SIV infection significantly reduced the cytotoxic capacity of these cells. Furthermore, SIV infection also increased the IL-15 stimulation threshold required to significantly improve NK cell cytotoxicity when compared with uninfected and vaccinated macaques. Similarly, we also evaluated the impact of vaccination and SIV infection on Fc- and cytokine-mediated NK cell effector responses. Interestingly, NK cells from vaccinated and SIV-infected macaques displayed similar response levels to both types of stimulation. Collectively, our findings show that early set point SIV viremia significantly impairs the cytotoxic potential of circulatory NK cells.

## Materials and Methods

### Animals, Immunization, SIV Challenge, and Sample Collection

Assays used viably frozen peripheral blood mononuclear cells (PBMCs) obtained from eight rhesus macaques enrolled in a previous vaccine study (17). As shown in Table 1, macaques from this previous study were primed twice with multigenic SIV plasmid DNA with or without IL-12 DNA or IL-15 DNA at weeks 0 and 4. Next, macaques received a single dose of Ad5hr-SIV recombinants at week 12 and were further boosted with SIV proteins in MPL-SE adjuvant at weeks 24 and 36. Finally, macaques were challenged intrarectally with 10 50% monkey infectious doses of SIV_mac251_. Animals were housed at Advanced BioScience Laboratories, Inc. (ABL, Kensington, MD, USA) and maintained according to institutional Animal Care and Use Committee guidelines and the NIH Guide for the Care and Use of Laboratory Animals. Blood samples were collected by venipuncture of anesthetized animals and PBMCs were obtained by centrifugation on Ficoll-Paque PLUS gradients (GE Healthcare, Piscataway, NJ, USA). PBMCs were frozen in 90% fetal bovine serum (Invitrogen, Carlsbad, CA, USA), 10% dimethyl sulfoxide (Sigma-Aldrich, St. Louis, MO, USA) and stored in liquid nitrogen until use. Upon thawing, good cellular viability (>85% live cells) with trypan blue viability dye was observed. Cells were washed and re-suspended in R-10 medium (RPMI 1640 containing 10% FBS, 2 mM l-glutamine, 1% non-essential amino acids, 1% sodium pyruvate, and antibiotics).

**Table 1:** **Summary of immunization regimen of macaques used in this study**.

Immunization group	Weeks 0 and 4 (IM)	Week 12 (IT)	Weeks 24 and 36 (IM)	Week 48 (IR)
1. DNA (*n* = 7)	DNA-SIV^a^ plasmids + control DNA	Ad5hr-SIV^b^ recombinants	SIV proteins^c^	SIV_mac251_^d^
2. DNA/IL-12 (*n* = 7)	DNA-SIV plasmids + DNA/IL-12	Ad5hr-SIV recombinants	SIV proteins	SIV_mac251_
3. DNA/IL-15 (*n* = 7)	DNA–SIV plasmids + DNA/IL-15	Ad5hr-SIV recombinants	SIV proteins	SIV_mac251_

*^a^Three DNA-SIV plasmids separately encoding SIV_mac239_env, SIV_mac239_gag, and SIV_mac239_rev/nef were administered together by the indicated route at 2.5 mg of DNA per dose. IM, intramuscular*.

*^b^Three Ad5hr-SIV recombinants separately encoding SIV_smH4_ env/rev; SIV_mac239_ gag; and SIV_mac239_ nef_Δ1–13_ were administered at 5 × 10^8^ pfu/recombinant/route. IT, intratracheal*.

*^c^SIV_mac251_ gp140; and SIV_mac239_ Nef proteins were given at a dose of 100 and 50 μg, respectively, in MPL-SE adjuvant*.

*^d^Challenge administered at 10 50% monkey infectious doses. IR, intrarectal*.

### 721.221 Cell Killing Assays

To evaluate the cytotoxic potential of circulatory NK cells, a flow cytometry-based killing assay was used. 721.221 target cells, which lack MHC-Class I, were fluorescently labeled with 2.5 μM PKH-26 red labeling (Sigma-Aldrich) and 2.5 μM CFDA-SE (Molecular Probes, Carlsbad, CA, USA) in order to separate them from non-fluorescent effector cells. Target cells were washed twice and plated in R-10 medium at a final concentration of 10,000 per well in V-bottom 96-well plates. Effector PBMCs were thawed a day prior to running the assays and cultured in media alone or in the presence of recombinant rhesus IL-15 (NIH/NCRR Resource for Non-human Primate Immune Reagents, Atlanta, GA, USA). After incubation, effector PBMCs were washed and then added at the indicated effector-to-target (E:T) ratios to a final volume of 200 μl. Plates were incubated at 37°C for 4 h. After incubation, cells were labeled with 0.5 μl of aqua LIVE/DEAD viability dye (Invitrogen) in 100 μl PBS per well. Plates were washed twice with PBS and finally re-suspended in 200 μl of a 2% PBS–paraformaldehyde solution. Plates were stored at 4°C until acquisition on a LSRII machine equipped with a high throughput system (BD Biosciences, San Jose, CA, USA). Specific killing was measured by incorporation of the Aqua LIVE/DEAD viability dye in CFSE^+^PKH^+^ target cells. Target cells cultured in the absence of effector cells were used as negative controls to correct for background levels of dead cells.

### NK Cell Stimulation Assays

NK cells were activated using either cytokines, Fc-receptor crosslinking or by co-culture with NK-sensitive 721.221 target cells. PBMCs (1–1.5 × 10^6^) were stimulated in 0.5 ml of R-10 medium in 5 ml Flow Cytometry tubes. Cytokine stimulations were done for 24 h with the indicated concentrations of recombinant macaque IL-15 and IL-18 (both from the NIH/NCRR Resource for Non-human Primate Immune Reagents), and recombinant human IL-12p70 (Peprotech, Rocky Hill, NJ, USA). Fc-receptor crosslinking and 721.221 cell stimulation were performed for 6 h by adding 4 μl of an APC-Cy7 anti-CD16 antibody (BD Biosciences) or 721.221 cells at a 5:1 E:T ratio, respectively. NK cell activation by crosslinking of CD16 surface receptors using fluorochrome-conjugated anti-CD16 antibodies was performed, as previously described (18). A fluorochrome-matched isotype control antibody was used as a negative control. PMA/Ionomycin (eBioscience, San Diego, CA, USA) was used as a positive control. In all stimulation conditions, BD GolgiPlug, BD GolgiStop, and an Alexa Fluor 647 anti-CD107a antibody (eBioscience) were added at the manufacturer’s recommended concentrations for the last 5 h of culture. Cells were subsequently washed and the expression levels of CD107a and IFN-γ were measured by Flow Cytometry. Background levels of CD107a and IFN-γ expression by non-stimulated PBMCs were subtracted from specific responses.

### Flow Cytometry Antibodies

Anti-human fluorochrome-conjugated monoclonal antibodies known to cross-react with rhesus macaque antigens were used, including FITC anti-CD20 (2H7), APC-Cy7 anti-CD16 (3G8), V450 anti-IFN-γ (B27), Alexa Fluor 700 anti-CD3 (SP34-2), and PerCP-Cy5.5 anti-CD8 (SK1), all from BD Biosciences; eFluor 660 anti-CD107a (eBioH4A3) from eBioscience; PE anti-NKG2A (Z199) from Beckman Coulter (Fullerton, CA, USA); QDot605 anti-CD8 (3B5), and the Yellow and Aqua Live/Dead viability dyes from Invitrogen; and QDot605 anti-CD4 (19Thy-5D7) from the NIH Non-human Primate Reagent Resource (Boston, MA, USA). Cells were stained for specific surface molecules, fixed and permeabilized with a Cytofix/Cytoperm Kit (BD Biosciences), and then stained for specific intracellular molecules. At least 250,000 singlet events (PBMCs) were acquired on a LSR II (BD Biosciences) and analyzed using FlowJo Software (Treestar Inc., Ashland, OR, USA). For all samples, gating was established using a combination of isotype and fluorescence-minus-one controls.

### Statistical Analysis

Data were analyzed as described in figure legends using Prism (v6.01, GraphPad Software). A *p* value ≤0.05 was considered statistically significant.

## Results

### Immunological and Virological Characteristics of Samples Used in This Study

**Figure 1:**
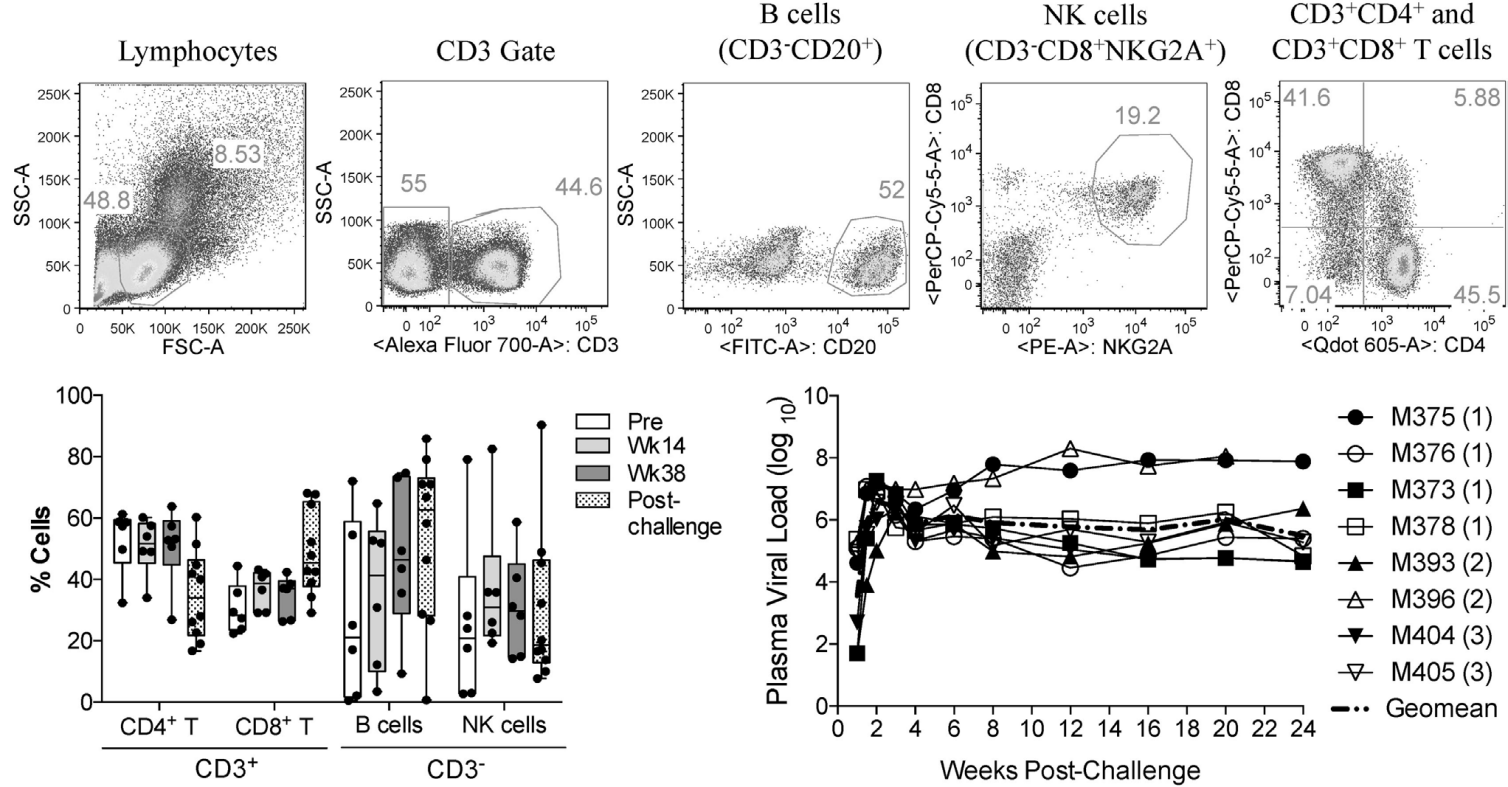
**Immune cell subsets and viral load status of macaque samples used in the present study**. Frozen PBMCs from previously vaccinated and SIV_mac251_-challenged macaques were thawed and stained with fluorochrome-conjugated monoclonal antibodies. **(A)** Gating strategy used to identify the proportional abundance of B cells, NK cells, and CD4^+^ and CD8^+^ T cells in samples. **(B)** Distribution of these immune subsets before vaccination (Pre), during vaccination (weeks 14 and 38) and 8–12 weeks after SIV_mac251_ intrarectal challenge (post-challenge) as determined by flow cytometry. Data are shown as minimum to maximum boxes with all data points represented. **(C)** Post-challenge viral loads in the eight macaques were used in the present study. Viral load data were taken from the previous report of Demberg et al. (17), and Vaccination group (described in Table 1) for each macaque is indicated in parenthesis.

In this study, we investigated the effects of vaccination and SIV infection on the functionality of circulatory NK cells (CD3^−^CD8^+^NKG2A^+^ lymphocytes). Samples used here had been viably frozen as part of a previous vaccination study (17). Although no protection from acquisition was observed in the previous study, samples were available from different time-points before and after challenge (pre-immunization, 14 and 38 weeks post-vaccination, and 8 and 12 weeks post-challenge). Table 1 describes the components of each vaccination group. Given that in the prior study, there were no observed differences in cellular or humoral immune responses between animals in each vaccination group (17), we combined available samples from vaccinated animals into a single group. Upon thawing of each frozen PBMC sample, immune cell composition was evaluated by measuring the proportional abundance of CD4 (CD3^+^CD4^+^), CD8 (CD3^+^CD8^+^), B (CD3^−^CD20^+^), and NK cells (CD3^−^CD8^+^NKG2A^+^) by flow cytometry (Figure 1A). As shown in Figure 1B, no significant changes in immune cell composition were observed in samples during vaccination or after infection. Figure 1C shows the plasma viral loads post-challenge for the eight PBMC samples used in the present study. To increase the sample size post-SIV challenge, samples from weeks 8 and 12 post-challenge time-points were combined into a single post-challenge group.

### NK Cells from SIV-Infected Macaques Are Less Effective at Mediating Direct Cytotoxic Function

**Figure 2:**
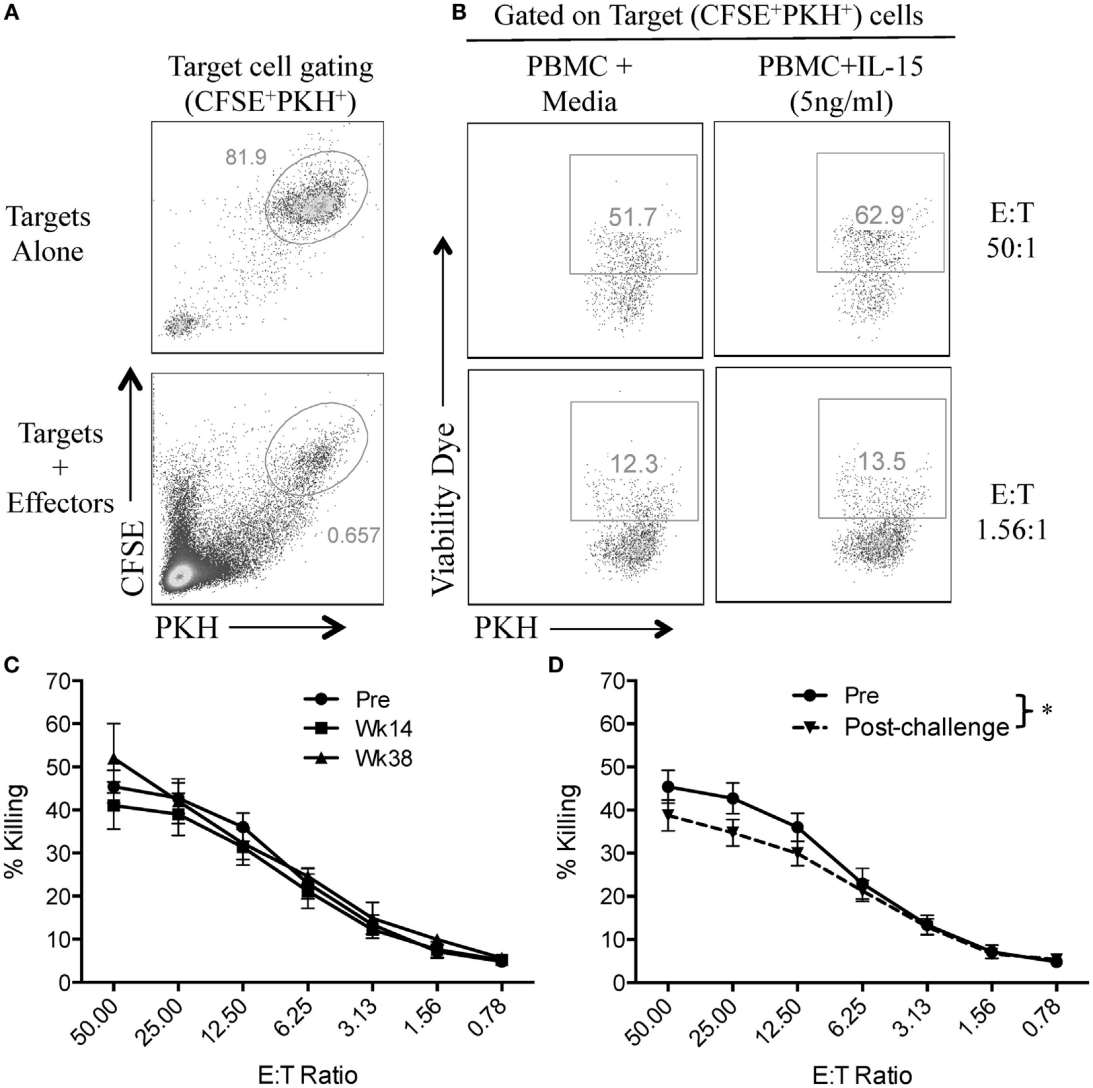
**SIV infection impairs natural cytotoxic capacity of rhesus macaque circulatory NK cells**. Frozen PBMCs were thawed and cultured overnight in media alone or in media supplemented with 5 ng/ml of recombinant rhesus macaque IL-15. PBMCs were then co-cultured with CFSE/PKH double-labeled 721.221 target cells at different effector-to-target cell ratios for 4 h. **(A)** Gating strategy used to differentiate 721.221 target cells (CFSE^+^PKH^+^) from unlabeled effector cells. **(B)** Killing of CFSE^+^PKH^+^ target cells as measured by the incorporation of the aqua amine-reactive dye. **(C,D)** 721.221 target cell killing by PBMCs from vaccination **(C)** and post-challenge **(D)** time-points as compared with PBMCs obtained pre-vaccination (Pre). Effector cells used in C and D were rested overnight in media alone. Data reported are means ± SEM. *p < 0.05 indicates statistically significant differences between the indicated killing curves as determined by two-way ANOVA.

In order to assess if vaccination or SIV infection had an effect on NK cell function, we first evaluated the capacity of circulatory NK cells to mediate natural cytotoxicity against MCH-1-devoid 721.221 cells. For this, we adapted a previously used flow cytometry-based killing assay and double-labeled 721.221 target cells with CFSE and PKH (Figure 2A) (13). This 721.221 cell killing assay allowed us to evaluate the cytotoxic potential of NK cells that had been incubated in the presence or absence of exogenous IL-15 at different target-to-effector cell ratios (Figure 2B). As shown in Figure 2C, no significant differences were observed in the cytotoxic capacity of NK cells in vaccinated macaques when compared with pre-immunization samples. On the other hand, we observed a significant reduction in NK cell cytotoxic function when pre-immunization samples were compared with post-challenge samples (Figure 2D).

### High Doses of IL-15 Pre-Activation Are Required to Rescue Cytotoxic Function in NK Cells from SIV-Infected Macaques

**Figure 3:**
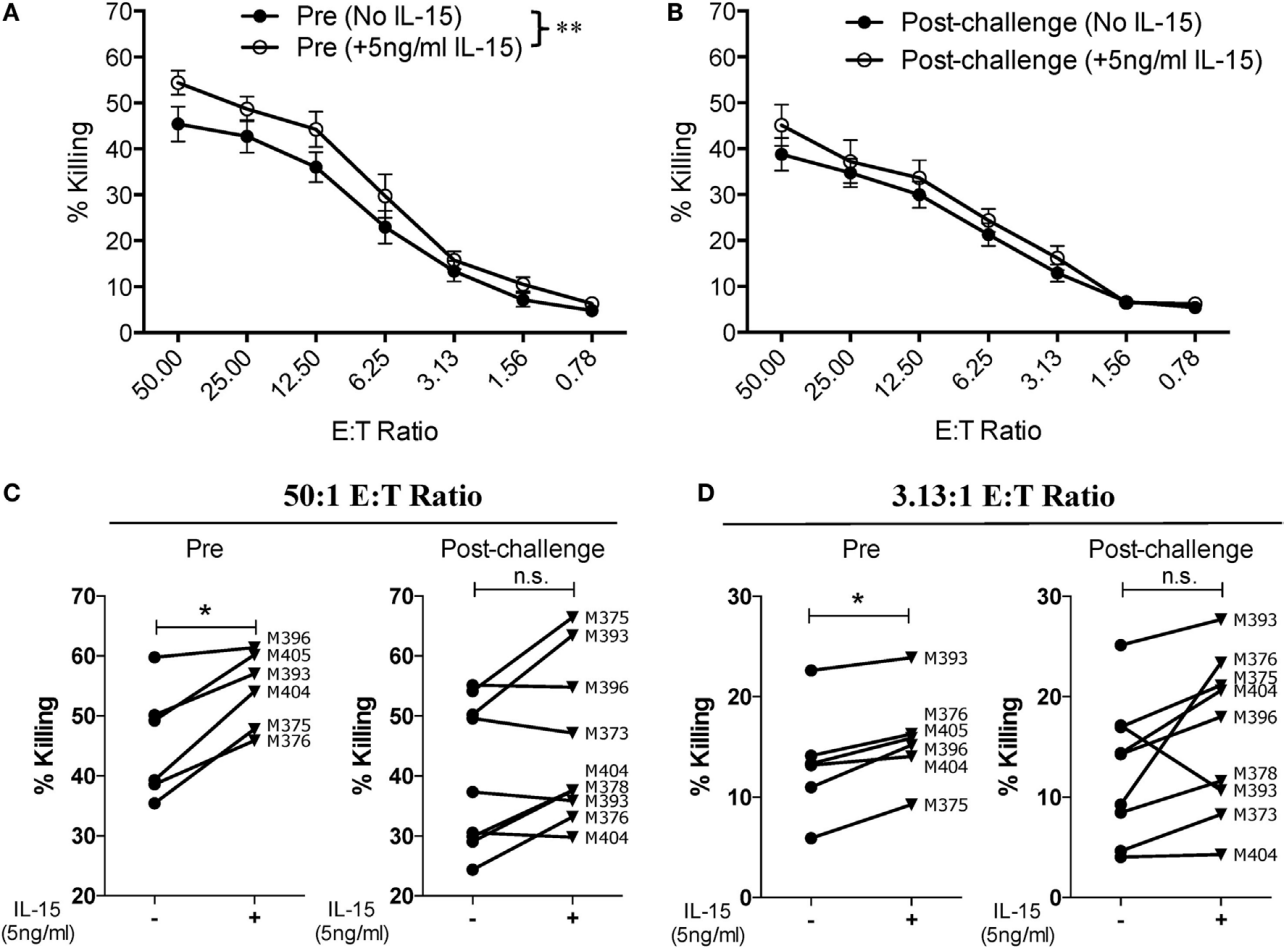
**Low dose of IL-15 pre-treatment does not improve cytotoxic potential of NK cells from SIV-infected macaques**. Frozen PBMCs were thawed and cultured overnight in media alone or in media supplemented with 5 ng/ml of recombinant rhesus macaque IL-15 before being used in 721.221 killing assays. **(A,B)** Comparison of killing by PBMCs from pre-vaccination **(A)** and post-challenge **(B)** time-points. Data reported are means ± SEM. **p < 0.01 indicates statistically significant differences between the indicated killing curves as determined by two-way ANOVA. **(C,D)** Impact of IL-15 pre-treatment on each type of effector cell at either a high [50:1 **(C)**] or a low [3.13:1 **(D)**] effector-to-target cell ratio. For these assays, six pre-vaccination samples were available and nine post-challenge samples were available (both weeks 8 and 12 post-challenge samples from macaque 393 were available). *p < 0.05 indicates statistically significant differences between the two compared conditions as determined by Wilcoxon matched-pairs signed-rank test. n.s., not significant.

**Figure 4:**
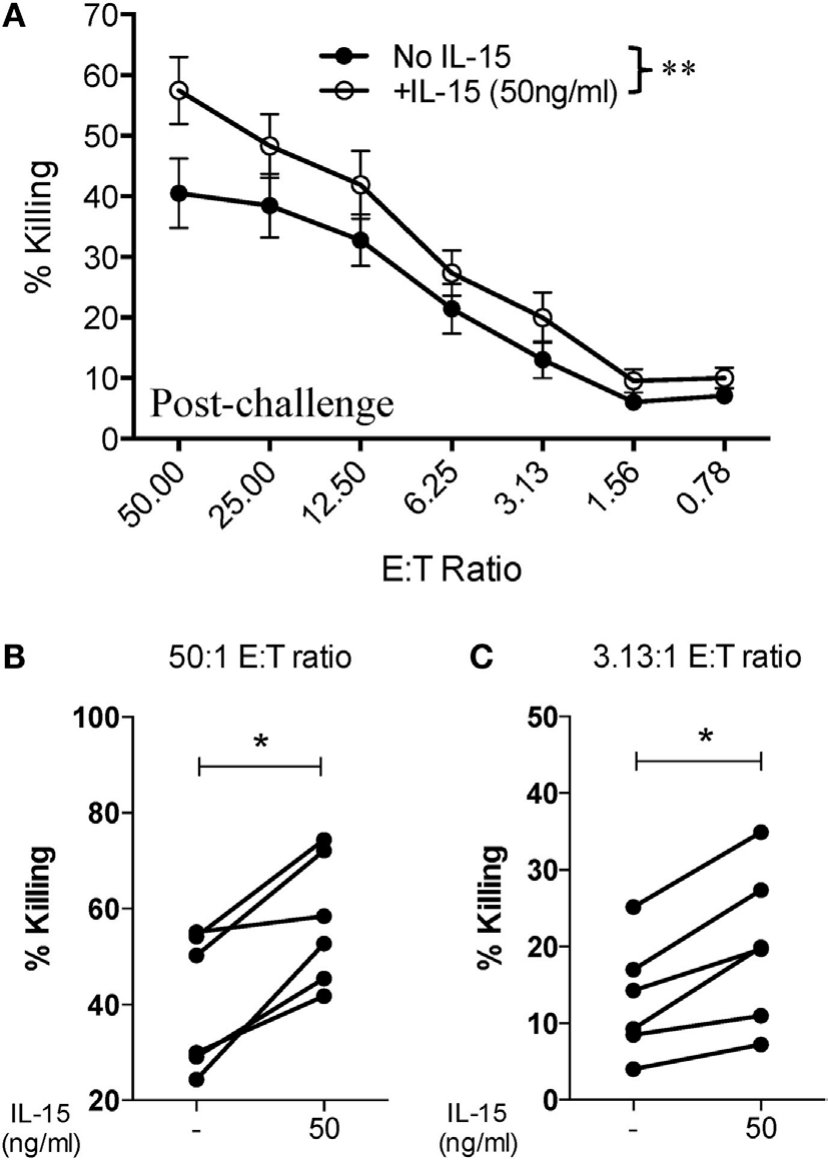
**Higher dose of IL-15 pre-treatment is required to improve cytotoxic potential of NK cells from SIV-infected macaques**. Frozen PBMCs obtained from six macaques at 8 or 12 weeks post-challenge were thawed and cultured overnight in media alone or in media supplemented with 50 ng/ml of recombinant rhesus macaque IL-15 before being used in 721.221 killing assays. **(A)** Killing of 721.221 target cells by PBMCs from SIV-infected macaques pre-cultured with or without 50 ng/ml of IL-15. Data reported are means ± SEM. **p < 0.01 indicates statistically significant differences between the indicated killing curves as determined by two-way ANOVA. **(B,C)** Impact of 50 ng/ml IL-15 pre-treatment on 721.221 target cell killing at either a high [50:1 **(B)**] or low [3.13:1 **(C)**] effector-to-target cell ratio. *p < 0.05 indicates statistically significant differences between the two compared conditions as determined by Wilcoxon matched-pairs signed-rank test.

Given that SIV infection impairs the natural cytotoxic potential of circulatory NK cells, we sought to determine if exogenous IL-15 pre-activation was able to reverse this impairment. To this end, we pre-activated PBMCs with 5 ng/ml of IL-15 for 18 h prior to their use. As shown in Figure 3A, IL-15 pre-activation at 5 ng/ml significantly increased the cytotoxic capacity of NK cells from uninfected macaques. On the other hand, pre-activation with 5 ng/ml of IL-15 did not improve the cytotoxic potential of NK cells from SIV-infected macaques (Figure 3B). The lack of responsiveness to IL-15 observed on NK cells from SIV-infected macaques was observed both at high (50:1, Figure 3C) and low (3.13:1, Figure 3D) E:T ratios. Next, we evaluated if a higher dose of IL-15 is needed to efficiently activate the cytotoxic potential of NK cells from SIV-infected macaques. For this purpose, we pre-activated PBMCs from SIV-infected macaques with 50 ng/ml of IL-15 prior to evaluating cytotoxic potential. As shown in Figure 4A, 50 ng/ml of IL-15 significantly increased the cytotoxic potential of circulatory NK cells from SIV-infected macaques. The improvement in cytotoxic potential observed by pre-incubating cells with 50 ng/ml of IL-15 was significant both at high (50:1, Figure 4B) and low (3.13:1, Figure 4C) E:T ratios.

### Vaccination Does Not Affect the Cytotoxic and Cytokine-Producing Function of NK Cells

**Figure 5:**
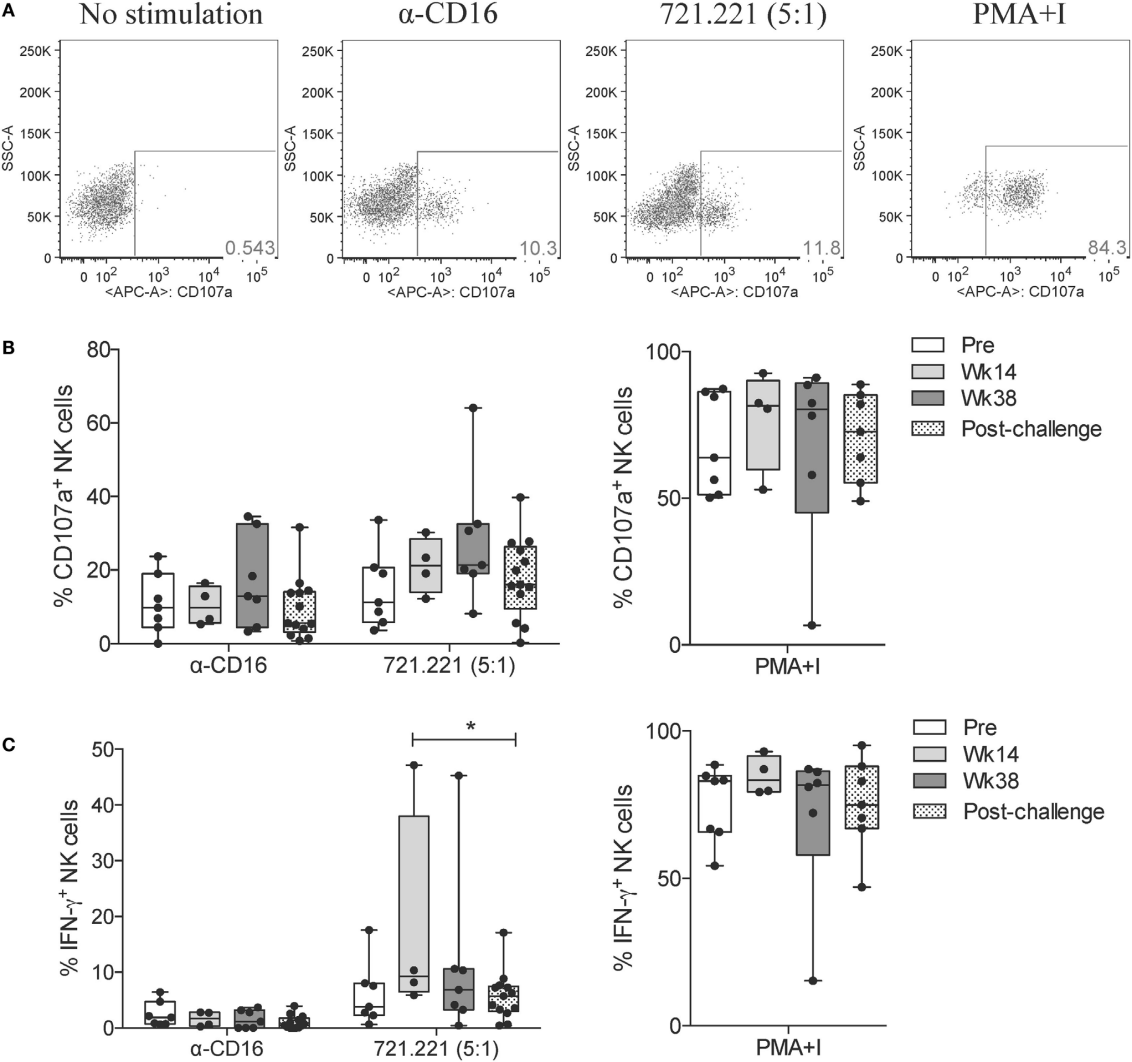
**NK cell cytotoxic responses through vaccination and after SIV infection**. Frozen PBMCs obtained over the course of vaccination and challenge were thawed, cultured overnight, and then stimulated for 5 h with anti-CD16 antibody, 721.221 cells (5:1 effector-to-target cell ratio) or PMA plus ionomycin. **(A)** Representative flow cytometry dot plots displaying the proportional abundance of CD107a^+^ cells within total NK cells (CD3^−^CD8^+^NKG2A^+^) for each stimulatory condition. **(B,C)** Expression of CD107a **(B)** and production of IFN-γ **(C)** by total NK cells in response to each stimulation was quantified at the indicated time-points. Values of non-stimulated samples and negative controls have been subtracted. Data are shown as minimum to maximum boxes with all data points represented. *p < 0.05 indicates statistically significant differences between the indicated time-points by Mann–Whitney test.

Having observed that SIV infection leads to an impairment in the killing capacity of circulatory NK cells, as measured by direct killing of MHC-I-devoid target cells, we evaluated the activation profile of circulatory NK cells in response to different cytotoxic and cytokine-producing stimulations. Surface expression of CD107a (LAMP-1) is a commonly used surrogate marker for cell degranulation and cytotoxicity (19). Stimulation of circulatory NK cells through CD16 crosslinking (α-CD16) or co-culture with 721.221 cells leads to NK cell degranulation and surface expression of CD107a (Figure 5A). When compared with pre-vaccination levels, a trend toward increased CD107a expression was observed at week 38 (end of vaccination protocol). This was observed both in response to CD16 crosslinking and 721.221 cell co-culture, but not in response to non-specific activation with PMA plus Ionomycin (Figure 5B). Given that CD16 crosslinking and 721.221 cell co-culture also upregulate IFN-γ production, we also evaluated this parameter in vaccinated and SIV-infected macaques. Although only minimal IFN-γ production was observed after CD16 crosslinking in both vaccinated and SIV-infected macaques, 721.221 cell co-culture induced detectable levels of IFN-γ (Figure 5C). A significant decrease in the production of IFN-γ by circulatory NK cells was observed when comparing samples from week 14 (after adenovirus vaccination) with post-infection (Figure 5C). No significant differences in IFN-γ production at any time-point were detected when stimulated with PMA plus Ionomycin (Figure 5C).

**Figure 6:**
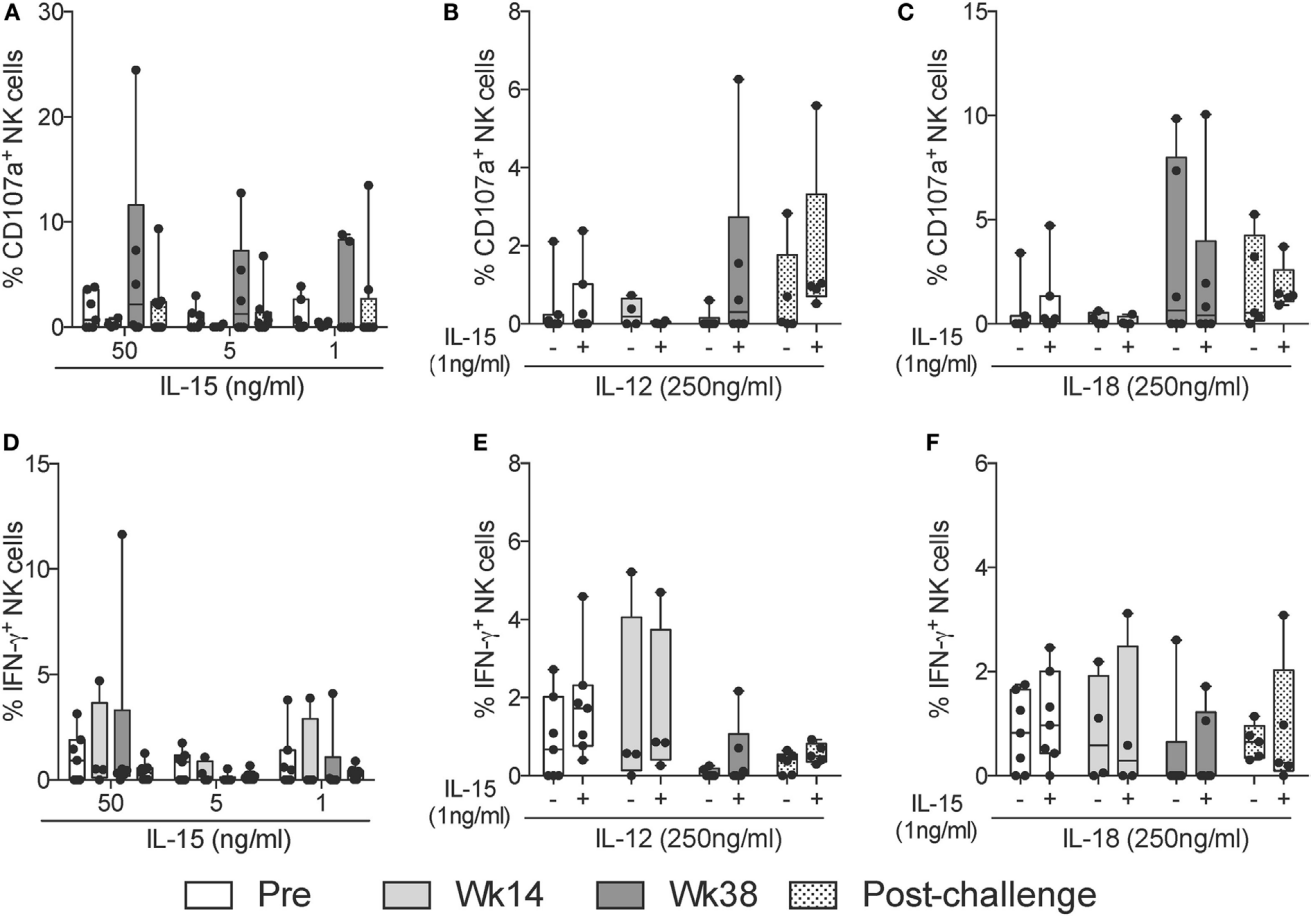
**NK cell cytokine-dependent responses through vaccination and after SIV infection**. Frozen PBMCs obtained over the course of vaccination and challenge were thawed and stimulated overnight in the presence of different concentrations and combinations of IL-15, IL-12, and IL-18. **(A–F)** Expression of CD107a (top row) or IFN-γ (bottom row) by total NK cells in response to high (50 ng/ml) and low (5 ng/ml) concentrations of IL-15 **(A,D)**; a high (250 ng/ml) dose of IL-12 in the presence (+) or absence (−) of a low (5 ng/ml) dose of IL-15 **(B,E)**; and a high (250 ng/ml) dose of IL-18 in the presence (+) or absence (−) of a low (5 ng/ml) dose of IL-15 **(C,F)**. Values of non-stimulated samples and negative controls have been subtracted. Data are shown as minimum to maximum boxes with all data points represented.

Given that NK cells can modulate the adaptive immune response by activating or inhibiting T cell responses (20), and due to their potential to rapidly respond to virus infection (21), we evaluated the capacity of circulatory NK cells from vaccinated and SIV-infected macaques to respond to different exogenous cytokine stimuli. We were particularly interested in responses to IL-15 stimulation given its pleiotropic effects and the major role played during inflammatory and protective immune responses (22). Interestingly, when directly stimulating circulatory NK cells with increasing concentrations of IL-15, we observed increased CD107a expression on week 38 samples (Figure 6A). These responses lacked statistically significance likely due to the small number of animals assayed. Other antigen-presenting cell-derived cytokines that play an important role in NK cell effector function are IL-12 and IL-18 (23, 24). To determine if circulatory NK cells from vaccinated and SIV-infected macaques have increased or reduced responsiveness to IL-12 and IL-18 stimulation, we performed stimulation assays with these two cytokines in the presence or absence of a low (1 ng/ml) IL-15 dose. When measuring CD107a expression, a trend toward increased expression was observed in response to IL-12 stimulation on week 38 and post-challenge samples only in the presence of IL-15 (Figure 6B). A similar trend, although independent of IL-15 addition, was observed after IL-18 stimulation (Figure 6C). Differently from CD107a expression, IFN-γ production was not significantly increased by IL-15, IL-12, or IL-18 in any of the concentrations and combinations tested (Figures 6D–F). It is important to mention that, in the present study, we did not evaluate NK cell functional responses in a subset-specific manner and, thus, the lack of responsiveness observed may be related to changes in the relative frequencies of NK cell subsets.

## Discussion

NK cell effector function becomes impaired during HIV and SIV infection (3). By taking advantage of viably frozen PBMC samples from a previous vaccination and challenge study (17), we observed that circulatory NK cells from SIV-infected macaques displayed decreased cytotoxicity. Moreover, NK cells from SIV-infected animals also required higher levels of IL-15 pre-stimulation in order to restore their natural cytotoxic potential. There has been disagreement in the literature on how HIV affects NK cell functionality. Some previous studies reported that viremic HIV-1 infection negatively impacts the cytolytic abilities of NK cells (25, 26), while others have shown that HIV elevates NK cell activity despite a reduction in cell number (27). Other studies have shown that NK cells from HIV-1 infected donors are able to form normal lytic conjugates with target cells; however, their ability to mediate lysis is inhibited (28). Similarly, the frequency of effector-to-target conjugates observed in HIV-1 patients is comparable to those observed in healthy controls (29).

Impairment of NK cell cytotoxicity can be caused by a change in surface expression of natural cytotoxicity receptors (NCRs). The expression of activatory NCRs, such as NKp46, NKp30, and NKp44, is known to be decreased among viremic patients. This defective NCR expression has been associated with a similar decrease in NK killing of different tumor target cells (30). IL-15 and IL-15 + sIL-15Rα conjugates have previously been shown to improve the cytotoxic potential of NK cells in various viral and neoplastic environments (31, 32). Particularly, long-term culture of NK cells in the presence of IL-15 and cells co-expressing IL-15Rα and 4-1BBL ligand (CD137L) has been shown to increase KIR, NKG2D, and NCR expression levels, thus improving cytotoxicity (33). It is plausible that overnight pre-activation of NK cells from SIV-infected rhesus macaques in high IL-15 concentrations similarly leads to a restoration of NCR surface expression and, therefore, partially improves natural cytotoxicity. Given that NCR expression in NK cells can be differentially regulated by cytokines (34), it would be of interest to evaluate the potential of other common-gamma chain cytokines, such as IL-2, IL-4, IL-7, IL-9, and IL-21, in restoring natural cytotoxicity in NK cells from SIV-infected macaques. As the present study utilized samples from the early set point phase of infection, evaluating the functional response of NK cells from long-term chronically infected rhesus macaques should provide insight into the role played by these cells during chronic infection. Moreover, while HIV infection can affect NCR expression on NK cells, dysfunction can also be caused by the upregulation of negative regulators, such as Tim-3 (35). It has previously been shown that 6 months of antiretroviral therapy can restore Tim-3 expression on NK cells and their responsiveness to inflammatory stimuli to normal levels (36). Whether combinatorial antiretroviral and cytokine-mediated therapy can further improve NK cell cytotoxic functionality and help reduce chronic viremia remains to be determined.

NK cell-dependent Fc-mediated functional responses are of great importance against pathogens as they serve as bridges between the innate and adaptive immune systems (37). Under the conditions tested in this study, we did not observe a significant change in the capacity of circulatory NK cells to respond to Fc-mediated stimulation after vaccination or infection. Future experiments evaluating cytotoxic, cytokine, and Fc-mediated functions of NK cells in SIV-infected animals that either succeed or fail to control chronic viremia may shed light into the role played by these cells and aid in the design of novel therapeutic and prophylactic approaches against SIV/HIV. Similarly, given the recent discovery of memory-like NK cell responses in mice, humans, and rhesus macaques (38–40), and given the apparent importance of pathogen-specific antibody-dependent responses in the expansion of memory-like NK cells (41), current efforts in our research group are directed toward determining whether SIV-specific vaccination leads to the development of memory-like responses in circulatory or tissue-resident NK cells.

## Author Contributions

DV-I and MR-G designed the study. DV-I and OY performed the experiments. TD supplied samples and reagents needed for this study. DV-I, OY, and MR-G analyzed and interpreted the data, and wrote the manuscript. All authors have read, reviewed, and edited the manuscript and agreed for submission to this journal.

## Conflict of Interest Statement

The authors declare that the research was conducted in the absence of any commercial or financial relationships that could be construed as a potential conflict of interest.
